# Supplementary material for: Accelerating inference in genomic and proteomic foundation models via speculative decoding
Source: bioRxiv. 2026 Jan 14:2026.01.13.699044. Preprint. [Version 1] doi: 10.64898/2026.01.13.699044 (PMC12871094; doi:10.64898/2026.01.13.699044)
Supplement: Supplement 1 [file NIHPP2026.01.13.699044v1-supplement-1.pdf]

## Supplementary Figures

bioRxiv preprint doi: https://doi.org/10.64898/2026.01.13.699044; this version posted January 14, 2026. The copyright holder for this preprint (which was not certified by peer review) is the author/funder, who has granted bioRxiv a license to display the preprint in perpetuity. It is made available under aCC-BY 4.0 International license.

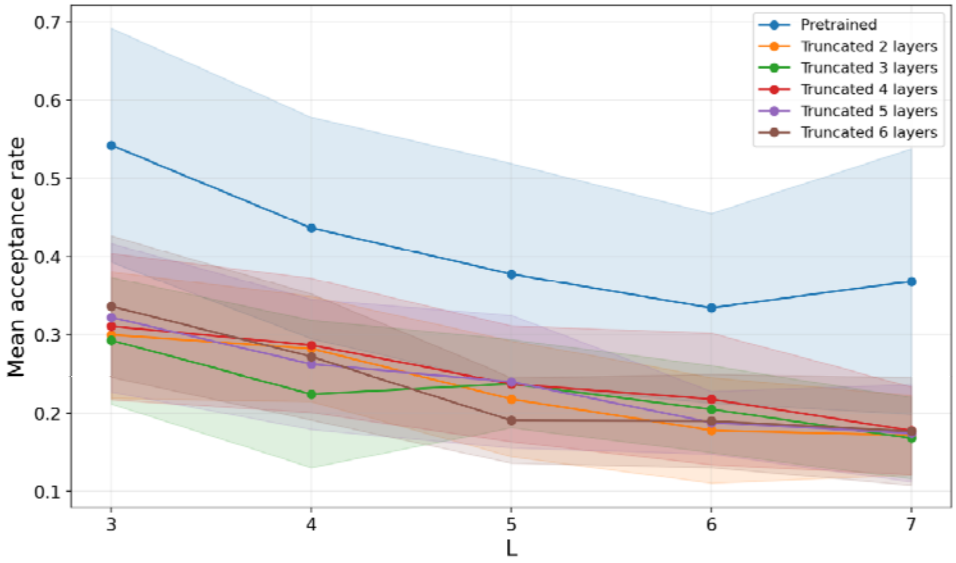


**Supplementary Figure 1: Mean acceptance rate for ProGen2 speculative decoding across draft architectures.** Mean draft-token acceptance rate as a function of speculation window size *L* for ProGen2, comparing a pretrained ProGen2-small draft with truncated drafts derived from the ProGen2-xlarge target using the first 2–6 Transformer layers. Lines show the mean over runs and shaded regions indicate variability across configurations.

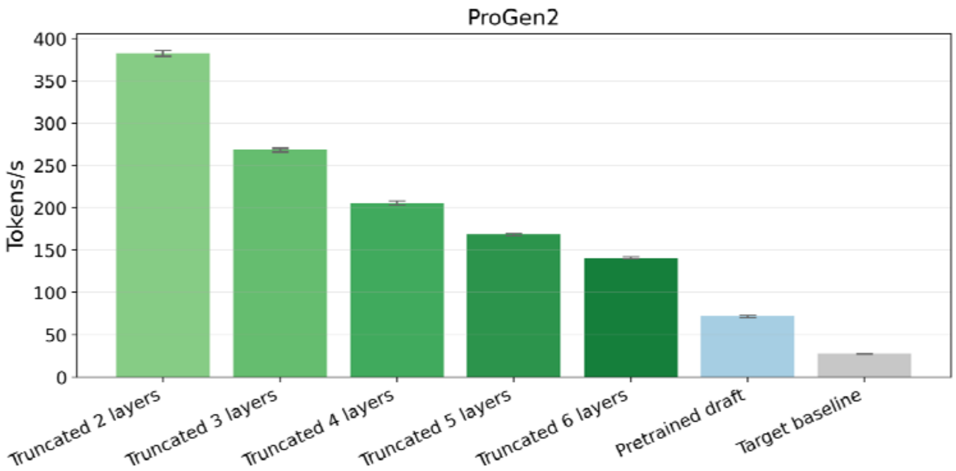


**Supplementary Figure 2: Throughput of ProGen2 speculative decoding for different models.** Mean decoding throughput (tokens per second) for ProGen2 when using truncated drafts with 2–6 Transformer layers, a pretrained ProGen2-small draft, and the KV-cached

bioRxiv preprint doi: https://doi.org/10.64898/2026.01.13.699044; this version posted January 14, 2026. The copyright holder for this preprint (which was not certified by peer review) is the author/funder, who has granted bioRxiv a license to display the preprint in perpetuity. It is made available under aCC-BY 4.0 International license.

ProGen2-xlarge target-only baseline. Bars show mean tokens/s over repeated runs and error bars denote variability across runs.

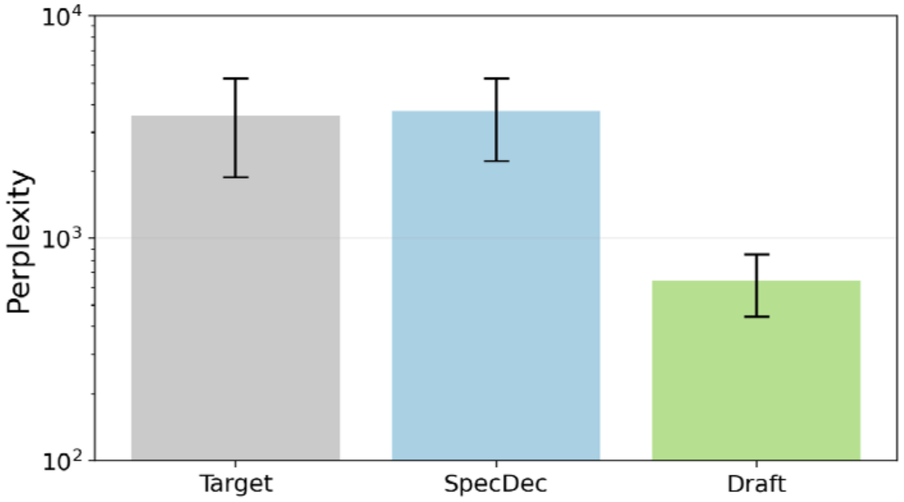


**Supplementary Figure 3:** Draft-model perplexity comparison for DNAGPT. Vertical bars (log scale) show mean suffix perplexity when sequences generated by the three DNAGPT decoders are rescored under the DNAGPT-0.1B draft model: “Target” denotes target-only decoding, “SpecDec” the probabilistic speculative decoding pipeline, and “Draft” the draft-only decoder. Error bars indicate standard deviation across generated suffixes.

## Supplementary Tables

**Supplementary Table 1: Sequences / Prompts used for the experiments on the three models.**

[Sequences / Prompts](#)
